# The relevance of long head biceps degeneration in the presence of rotator cuff tears

**DOI:** 10.1186/1471-2474-11-191

**Published:** 2010-08-27

**Authors:** Stefan Lakemeier, Johannes JA Reichelt, Nina Timmesfeld, Susanne Fuchs-Winkelmann, Juergen RJ Paletta, Markus D Schofer

**Affiliations:** 1 Department of Orthopedics and Rheumatology, University Hospital Marburg, Germany; 2 Institute of Medical Biometry and Epidemiology, Phillips-University Marburg, Germany

## Abstract

**Background:**

Long head biceps (LHB) degeneration in combination with rotator cuff tears can be a source of chronic shoulder pain. LHB tenotomy is an approved surgical procedure for pain reduction and improvement of joint function, however, the pathophysiology of LHB degeneration is not fully understood. In the literature, neoangiogenesis in tendon tissue has previously been shown to be associated with tendon degeneration. Vascular Endothelial Growth Factor (VEGF) is an important inducer of neoangiogenesis. The hypotheses are first that an elevated VEGF expression and vessel density can be found in degenerated LHB tissue and second that there is a relation between VEGF expression, vessel density and the different types of rotator cuff tears.

**Methods:**

LHB samples of 116 patients with degenerative rotator cuff tears were harvested during arthroscopic tenotomy. The samples were dehydrated and paraffin embedded. VEGF expression was determined using immunohistochemistry. Vessel density and vessel size were determined on Masson-Goldner stained tissue sections. On the basis of intraoperative findings, patients were assigned to 4 different groups (control group, partial thickness rotator cuff tear, full thickness rotator cuff tear and cuff arthropathy). Partial thickness rotator cuff tears were classified according to Ellman grade I-III, full thickness rotator cuff tears according to Bateman's classification (grade I-IV). The control group consisted of eight healthy tendon samples.

**Results:**

VEGF expression in the LHB was significantly higher in the presence of rotator cuff tears than in healthy tendons (p < 0.05) whereas vessel density and vessel size were significantly higher in the LHB of patients with cuff arthropathy (p < 0.05). Furthermore, there was significantly higher VEGF expression in LHB samples from patients with articular-sided compared to bursal-sided partial thickness rotator cuff tears (p < 0.05). No significant dependence was found between VEGF expression, vessel size and vessel density in LHB of patients with full thickness rotator cuff tears and the extent of the cuff tear following Bateman's classification.

**Conclusion:**

Elevated VEGF expression can be detected in degenerated LHB tissue. The quantity of VEGF expression and vessels are related to the extent of LHB degeneration.

## Background

Abnormalities of the long head biceps tendon (LHB) are often associated with rotator cuff tears and may be a reason for persisting shoulder pain [1-3]. The arthroscopic tenotomy of the degenerated LHB is a standard procedure in orthopedic shoulder surgery and leads to significant pain release [4,5]. The LHB degeneration can be diagnosed clinically and via Magnetic Resonance Imaginary (MRI) based on alterations of tendon diameter and signal abnormalities [6,7]. Whereas tendinopathy has been studied extensively in the supraspinatus, Achilles, patellar and extensor carpi radialis brevis tendons, there is much less information on tendon degeneration of LHB [8-10]. The anatomy of the LHB is unique. The proximal part of the tendon is located intraarticular. LHB degeneration may occur primarily or can be due to changes of the glenohumeral joint or the surrounding musculature [11]. The extraarticular part is protected under the pectoralis major and subjected primarily to tensional strain [12]. In the literature, studies examining the histopathological findings of the intraarticular portion of LHB in active patients are rare. Longo et al. performed a study on 51 ruptured LHB obtained at the time of tenotomy. They demonstrated that the ruptured tendons exhibited marked histopathologic changes in comparison to cadaveric control tendons which had little pathologic change [13]. Joseph et al. compared the histological and molecular changes of the intraarticular and extraarticular part of the LHB [14]. The intraarticular LHB exhibited significantly greater histological tendinopathy inclusive of increased proteoglycan and decreased organization of collagen fibers. Vascular Endothelial Growth Factor (VEGF) is a growth factor capable of stimulating of endothelial cells and vessels to invade hypovascularized tendon areas [15]. Pufe et al. as well as Peterson et al. could detect higher concentrations of VEGF in degenerative Achilles tendon tissue compared with healthy adult tendon tissue [16,17]. In another study, Petersen et al. could further demonstrate, that angiogenesis contributes to the repair and remodeling of the degenerated tendon but may also weaken the mechanical stability of the tendon by invasion of the endothelial cells [15].

The purpose of this study was to evaluate whether VEGF and angiogenesis can be observed in the degeneration of the LHB. Second, we aimed to examine the potential correlation between the extent of vessel density and VEGF expression in the degenerated LHB and the type of rotator cuff tear.

## Methods

### Patients

A total of 116 patients (55 male, 61 female) was recruited and included in this study. Full ethical approval was granted for the project by the ethics committee of the Medical Faculty at the University of Marburg, Germany. Preoperative written informed consent was obtained in all cases. 108 patients had a rotator cuff tear indicating a need for arthroscopic shoulder surgery and LHB tenotomy. LHB tissue specimens were harvested by arthroscopic tenotomy during arthroscopic shoulder operations performed by the senior author. The control group consisted of eight trauma patients with humeral head fractures. In these patients, LHB samples were harvested during humeral head prosthesis implantation. In every patient in the control group, the rotator cuff was intraoperatively seen to be faultless.

All patients were assigned to the following four groups on the basis of intraoperative findings:

• Group I: Patients with no shoulder pathology (control group)

• Group II: Patients with partial thickness rotator cuff tear

• Group III: Patients with full thickness rotator cuff tear

• Group IV: Patients with cuff arthropathy [18]

Partial thickness rotator cuff tears were classified using the classification of Ellman (grade I-III) [19] and were divided into articular-sided (A) and bursal-sided (B) partial thickness rotator cuff tears. Full thickness rotator cuff tears were diagnosed according to Bateman's classification (grade I-IV) [20]. Additional file 1 gives a detailed overview of the included patients.

### Specimen preparation

After harvesting, the obtained LHB samples were immediately fixed in 4% formaldehyde for 24 hours, dehydrated in graded alcohol solution and cedarwood oil and embedded in paraffin. Sections were cut at 5 μm with a Leica microtome RM2055 (Bensheim, Germany) with a 40° stainless-steel knife. Histological standard staining was performed with Masson-Goldner staining kit (Merck, Dramstadt Germany) according to the manufacturer instructions.

### Histology

Histomorphometrical analysis was performed at a primary objective lens magnification of 5 fold using a Leica DM5000 and Quips analysis software (Leica Bensheim, Germany). In order to characterize the cells and to perform cell count fortyfold objective lens magnification was used. For differential stained sections, for example Masson Goldner stained sections, a tenfold objective lens magnification was used. The vessel number and size was determined by counting and measuring the vessels in three different areas of every specimen. The cell counting of immunohistological stained sections was performed by a fortyfold objective lens magnification. The percentage stands for the quotient of VEGF positive cells in relation to the total number of cells per section.

### Immunohistochemistry

For immunohistological staining of VEGF, the sections were rehydrated and incubated in citrate buffer (pH 6) at 90°C over a period of 10 min. After blocking with normal horse serum, the sections were incubated overnight (4°C, humidified chamber) with a monoclonal antibody against Vascular Endothelial Growth Factor (VEGF clone VG1; Millipore MAB3734, dilution 1:750).

Immunostaining was performed using a labeled streptavidin-biotin method (Dako REAL Detection System Peroxidase/DAB+), the staining reaction being based on 3,3'-diaminobenzidine (DAB). The stained slides were rinsed with distilled water and stained for 15 seconds with haemalaun as counterstain. Finally, the sections were rinsed with water and treated with graduated density alcohol and with xylol.

### Statistics

Analysis of variance (ANOVA) and a modified least-square difference (Bonferroni) test were used to evaluate the differences between the experimental and control sites and between the different groups. Data are given as means ± standard error of measurements (SEM). The level of significance was set at p < 0.05. The Spearman-Rho test was used for the description of potential correlations.

## Results

### Group I (Control group)

**Figure 1:**
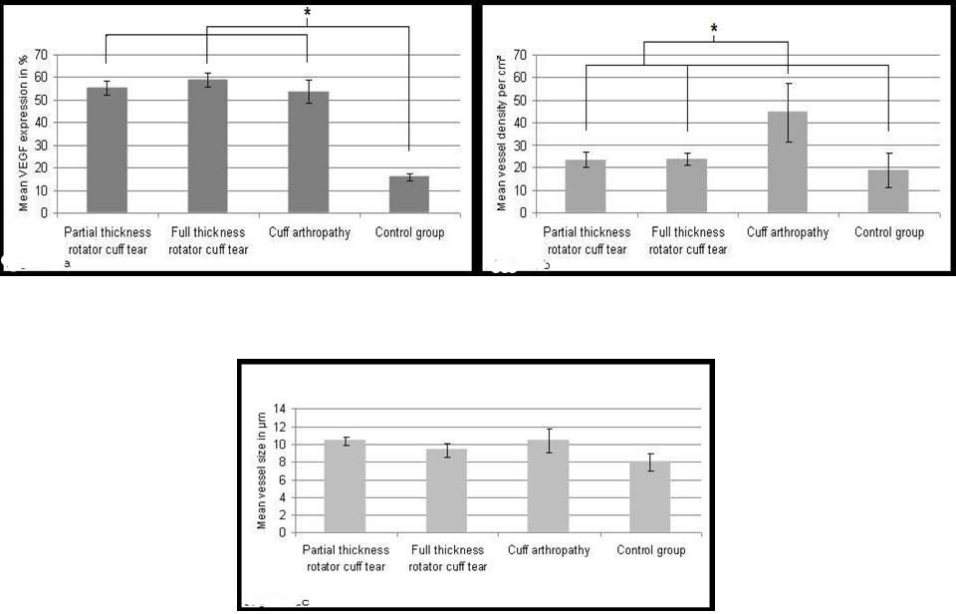
**a-c: Mean values for percentage of VEGF expression (a), vessel density in vessels/cm^2 ^(b) and vessel size in μm (c) for the different groups**.

The control group consisted of 8 patients (4 male, 4 female). Mean age was 56 (37-69) years. Mean VEGF expression was 15.87 ± 1.61% in control specimens. Mean vessel density was 18.62 ± 7.69 vessels/cm^2 ^and mean vessel size was measured with 7.99 ± 0.95 μm. Mean VEGF expression, vessel density and vessel size of the different groups are listed in figure 1.

### Group II (Partial thickness rotator cuff tears)

**Figure 2:**
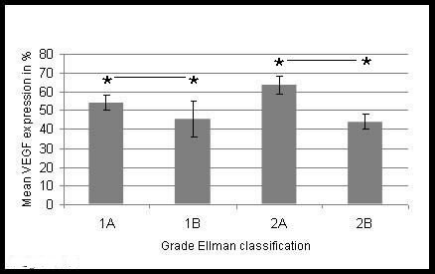
**Mean values for percentage of VEGF expression in articular-sided (A) and bursal-sided (B) partial thickness rotator cuff tears grade Ellman I and II**.

There were 48 patients (24 male, 24 female) with partial thickness rotator cuff tears. Mean age was 61 (39-78) years. 33 patients were classified as grade Ellman I. 28 partial thickness rotator cuff tears were situated articular-side and 5 tears were found on the bursal side of the rotator cuff. 15 patients were classified Ellman II (7 tears articular-sided, 8 tears bursal-sided). No patient was classified Ellman III. In this group mean VEGF expression was 52.83 ± 3.05%, mean vessel size was 10.40 ± 0.51 μm and mean vessel density was 23.37 ± 3.51 vessels/cm^2^. Significantly, more VEGF expression was found in articular-sided than in bursal-sided partial thickness rotator cuff tears (p < 0.05) (figure 2). Compared with the control group, significantly more VEGF expression was found (p < 0.05). Mean vessel size and mean vessel density were not significantly increased in comparison with the control group (p > 0.05).

### Group III (Full thickness rotator cuff tears)

This group consisted of 42 patients (20 male, 22 female). Mean age was 67 (55-80) years. The number of patients classified in the different Bateman grades, the mean VEGF expression, mean vessel size and mean vessel density are listed in additional file 2. VEGF expression was significantly increased compared with the control group (p < 0.01). Mean vessel density and mean vessel size were not significantly elevated (p < 0.05). Furthermore, there was no statistical significant correlation between VEGF expression, mean vessel number, mean vessel density and increasing Bateman grade (p > 0.05).

### Group IV (Cuff arthropathy)

In 18 patients (7 male, 11 female), cuff arthropathy was diagnosed. The diagnosis was made in cases of irreparable massive rotator cuff tear combined with complete chondral destruction. Patient age was 70 (51-87) years in average. Mean vessel size and mean vessel density were significantly higher than in the control group (p < 0.05). Compared with groups II and III, mean vessel density was significantly elevated (p < 0.01). VEGF expression was significantly higher than in the control group p < 0.05) but not significantly elevated in comparison with groups II and III (p > 0.05).

No statistical correlation could be found between VEGF expression (p = 0.94), vessel density (p = 0.81), vessel size (p = 0.32) and age of the 116 included patients.

**Figure 3:**
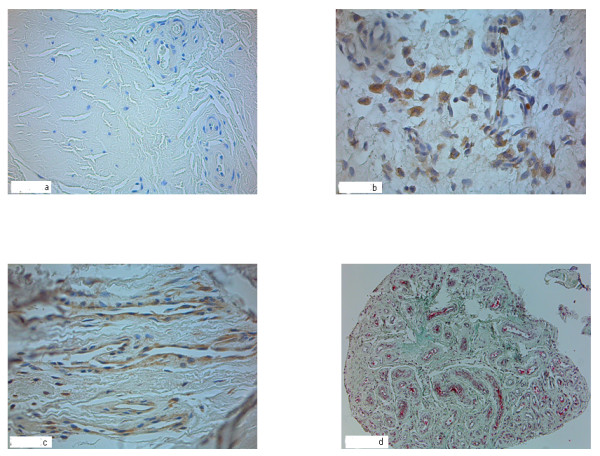
**a-d: LHB tendon sections after staining with VEGF antibody**. Fortyfold magnification objective lens magnification (a-c). In the control group (a) no VEGF expression and little vessel density can be observed. Example for group II "partial thickness rotator cuff tears" (b) with more VEGF expression and minor vessel density. In the group "full thickness rotator cuff tears" VEGF expression is higher and many vessels are visible (c). Example for the group IV "Cuff arthropathy" (Tenfold objective lens magnification, Masson-Goldner staining) with a high number of vessels visible (d).

Examples for tendon sections in the different groups and with different straining against the different antibodies can be observed in figure 3a-d.

## Discussion

We were able to show that VEGF is expressed in degenerated LHB and leads to higher vessel density, whereas the angiogenetic peptide level is lower in healthy LHB. The process of LHB degeneration and its possible conjunction with rotator cuff tears remains controversial. In a cadaveric study on seven shoulders with rotator cuff tears and seven healthy shoulders, Carpenter et al. could not describe any structural or histopathologic differences of the particular LHB. Therefore, the authors assume that LHB appears to retain its material properties in the presence of rotator cuff tears [21]. In contrast to these findings Peltz et al. could demonstrate that in a rat model, LHB mechanical properties worsened over time in presence of multiple rotator cuff tendon tears [22]. They assume that LHB function as humeral head stabilizer is enhanced because the tendons that normally support those functions are weakened and the LHB is required to perform new functions with altered mechanical loading. LHB degeneration may be a result of normal age-dependent shoulder pathology as it occurs in the presence or absence of cuff tears. Rathbun and Macnab suggested a vascular insufficiency in the affected area of the LHB at the entrance to the biceps groove to be responsible for the degeneration of the tendon [23]. These findings are supported by Refior and Sowa who described, on the basis of cadaveric studies, a tendency towards LHB degeneration in the bicipital groove [11]. In contrast to these findings, a recently published theoretical model published by Peers et al. suggests that chronic tendon loading leads to mechanical trauma of the tissue with enduring microruptures of the tendon microvasculature [24]. These microvascular ruptures initiate a VEGF mediated vascular remodeling that becomes pathological over time. We found the highest density of vessels and the highest mean vessel size in LHB of patients with cuff arthropathy. High amounts of VEGF can be seen in both, patients with partial thickness rotator cuff tears and patients with full thickness rotators cuff tears. According to Peer's model, our findings indicate that LHB mechanical load is intensified in the presence of rotator cuff tears. The above mentioned model described by Peltz et al. and the finding that full thickness rotator cuff tears are responsible for distinctive glenohumeral instability whereas partial thickness tears cause dynamic instability especially in the mid-range and end-range of motion, support our assumption that LHB degeneration is secondary to the existence of rotator cuff tears [18,22,25].

The role of VEGF and its function in tendon degeneration are largely unknown. The original function of VEGF as a strong inductor of embryonic vasculogenesis and development was shown using gene knockout studies in mice [26]. Other authors state the pivotal role of VEGF in the angiogenesis of certain tumors e.g. glioblastomas, and other pathological conditions associated with high neovascularisation e.g. diabetic retinopathy, age-related macular degeneration or rheumatoid arthritis [27]. The role of LHB as a pain generator is well recognized in literature but pain mediation remains unclear [28]. In a study performed on Achilles tendon biopsies, Alfredson et al. found accompanying nerves around the neovessels [29]. The authors hypothesize that these findings could indicate that the area with neovascularisation is of importance for Achilles pain. It is unclear, if this model can be transferred to LHB.

Our second aim was to examine a potential correlation between the extent of rotator cuff tears, VEGF expression and vessel density in LHB tissue. We demonstrated a significant correlation between the presence of rotators cuff tears and the quantity of VEGF expression, vessel size and vessel density in LHB tissue.

In a study published recently, Ko et al. found out that articular-sided partial thickness rotator cuff tears are largely associated with intrinsic pathological changes of the shoulder joint, whereas bursal-sided tears are more associated with impingement syndrome and an underlying milder pathological change of the rotator cuff [30]. Our finding are in accordance with these results as we could show that VEGF expression was higher in tissue samples harvested from patients with articular-sided partial thickness rotator cuff tears than in bursal-sided partial rotator cuff tears. Hence, it seems likely that genesis of LHB degeneration is secondary to intraarticular pathological changes of the joint. Unfortunately, we did not have any patients with grade III partial thickness rotator cuff tears to support this hypothesis. However, further biomechanical research is needed for complete understanding of this relationship.

The development of rotator cuff tears is still a point of discussion. Some authors state that cuff tears develop due to mechanical damage of the tendon caused by subacromial impingement [31]. Others affirm that degeneration is generated because of partial tendon hypovascularisation or primary tendon degeneration [32]. However, consensus exists that partial thickness rotator cuff tears may develop into full thickness rotator cuff tears. On the basis of ultrasound findings, Gohlke indicated that the mean age of patients with degenerative full thickness rotator cuff tears is higher than that of patients with partial thickness rotator cuff tears what is in accordance to our findings [33]. A full thickness rotator cuff tear may progress to cuff arthropathy [34]. We could show that VEGF expression is high in groups II and III (partial thickness rotator cuff tear, full thickness rotator cuff tear) whereas the average vessel density is higher in the group IV which may be the result of high VEGF expression. Our findings indicate that LHB degeneration is related to the course of degenerative rotator cuff disease. Despite the fact that LHB degeneration and degenerative shoulder disease may develop concomitantly due to common ethiological factors, we think that our results suggest that the genesis of LHB degeneration follows degenerative shoulder disease. These findings have not been reported so far and need further research to be completely understood.

The present study has several limitations. Our control group is small compared to the other groups. As VEGF plays a pivotal role in the formation of certain tumors, it was not possible to accept tumor patients with indication for tumor prosthesis implantation or upper limb amputation. Therefore, we decided to take LHB samples of trauma patients with comminuted humeral head fractures and indication for humeral head prosthesis implantation. This indication is rare in young patients and the operation is performed mainly on the elderly. However, we were able to include 8 patients with healthy LHB and rotator cuff as control group into this study. Although we tried to find patients of matchable age for the control group the patients of the control group are in average younger than the patients in the other groups. If LHB degeneration was a normal result of aging, the factor age could explain our results in part. As there is no significant statistical correlation between patient age, VEGF expression, vessel size and vessel density, we are convinced that normal aging cannot be the main explanation for our findings. Furthermore, the difference in age is relatively small between the different groups.

Full thickness rotator cuff tears may develop into cuff arthropathy in the course of the disease so that many full thickness rotator cuff tears are combined with different levels of osteoarthritis of the shoulder. In the groups "Bateman I-IV" osteoarthritis was not measured but might influence the findings for VEGF expression. Furthermore every patient of group IV (cuff arthropathy) had a massive rotator cuff tear so that the two groups cannot be strictly separated and differentiation between the two groups is floating. This bias could be balanced by the high density of patients in each group.

## Conclusion

Our data shows that LHB degeneration is associated with an elevation of VEGF expression and increased vessel density. Furthermore, on the basis of our results and the results from previous studies, it seems likely that LHB degeneration is secondary to the development of rotator cuff tears and is aggravated over the course of degenerative shoulder disease.

## Competing interests

The authors declare that they have no competing interests.

## Authors' contributions

SL was the main composer of the manuscript, JJAR accomplished the histological and immunohistological testing, NT participated in the design of the study and performed the statistical analysis. JRJP was involved in the study design and the immunohistological examinations. SFW conceived of the study, and participated in its design and coordination and helped to draft the manuscript. MDS designed the study, performed the surgical interventions and obtained the LHB samples. All authors read and approved the final version of the manuscript.

## Pre-publication history

The pre-publication history for this paper can be accessed here:

http://www.biomedcentral.com/1471-2474/11/191/prepub

## Supplementary Material

Additional file 1 **Overview of the included patients**. Classification of their shoulder pathology and the mean values for vessel size, vessel density and VEGF expression in the different groups. Click here for file

Additional file 2 **Overview of the included patients suffering from full thickness rotator cuff tears**. Mean values for VEGF expression, vessel density and vessel size for the different grades of full thickness rotator cuff tears. Click here for file
