# Supplementary material for: The relevance of long head biceps degeneration in the presence of rotator cuff tears
Source: BMC Musculoskelet Disord. 2010 Aug 27;11:191. doi: 10.1186/1471-2474-11-191 (PMC2936349; doi:10.1186/1471-2474-11-191)
Supplement: Additional file 1 — Overview of the included patients. Classification of their shoulder pathology and the mean values for vessel size, vessel density and VEGF expression in the different groups. [file 1471-2474-11-191-S1.DOC]

| **Diagnosis** | **Number of patients** | **Number of patients male** | **Number of patients female** | **Mean age**  **(years)** | **Mean MMP 1 expression (percentage of positive cells) ± standard error** | **Mean MMP 3 expression (percentage of positive cells) ± standard error** | **Mean MMP 9 expression**  **(percentage of positive cells) ± standard error** |
| --- | --- | --- | --- | --- | --- | --- | --- |
| **Control group (group I)** | 6 | 3 | 3 | 56 (47-69) | 38.54 ± 7.5 | 27.01 ± 2.01 | 9.52 ± 5.09 |
| **Patte grade 1**  **(group II)** | 6 | 0 | 6 | 61 (55-68) | 74.00 ± 6.08 | 8.00 ± 2.65 | 20.00 ± 4.01 |
| **Patte grade 2**  **(group III)** | 10 | 4 | 6 | 65 (55-75) | 59.9 ± 6.98 | 9.40 ± 2.77 | 34.10 ± 2.62 |
| **Patte grade 3**  **(group IV)** | 17 | 8 | 9 | 69 (51-79) | 60.28 ± 3.07 | 7.28 ± 1.65 | 44.53 ± 4.57 |

Additional file 1: Title: Overview of the included patients.

Description: Classification of their shoulder pathology and the mean values for vessel size, vessel density and VEGF expression in the different groups.
