# Supplementary material for: The relevance of long head biceps degeneration in the presence of rotator cuff tears
Source: BMC Musculoskelet Disord. 2010 Aug 27;11:191. doi: 10.1186/1471-2474-11-191 (PMC2936349; doi:10.1186/1471-2474-11-191)
Supplement: Additional file 2 — Overview of the included patients suffering from full thickness rotator cuff tears. Mean values for VEGF expression, vessel density and vessel size for the different grades of full thickness rotator cuff tears. [file 1471-2474-11-191-S2.DOC]

| **Grade Bateman classification** | **Bateman I** | **Bateman II** | **Bateman III** | **Bateman IV** |
| --- | --- | --- | --- | --- |
| **Number of patients** | 3 | 15 | 11 | 13 |
| **Mean vessel density per cm²** | 13.69 **±**7.40 | 23.36 **±**4.56 | 19.02 **±**5.27 | 26.53 **±**4.36 |
| **Mean vessel size (µm)** | 12.11 **±**6.37 | 9.03 **±**1.35 | 9.81 **±**1.39 | 9.44 **±**1.20 |
| **Mean VEGF expression (% of positive cells)** | 35.58 **±**15.16 | 60.29 **±**5.23 | 59.50 **±**6.25 | 55.29 **±**3.81 |

Additional file 2: Title: Overview of the included patients suffering from full thickness rotator cuff tears. Description: Mean values for VEGF expression, vessel density and vessel size for the different grades of full thickness rotator cuff tears.
